# Coordinated Actions of FXR and LXR in Metabolism: From Pathogenesis to Pharmacological Targets for Type 2 Diabetes

**DOI:** 10.1155/2014/751859

**Published:** 2014-04-28

**Authors:** Lin Ding, Shuguang Pang, Yongmei Sun, Yuling Tian, Li Yu, Ningning Dang

**Affiliations:** Endocrinology Department, Jinan Central Hospital Affiliated to Shandong University, No. 105 Jiefang Road, Jinan, Shandong 250013, China

## Abstract

Type 2 diabetes (T2D) is the most prevalent metabolic disease, and many people are suffering from its complications driven by hyperglycaemia and dyslipidaemia. Nuclear receptors (NRs) are ligand-inducible transcription factors that mediate changes to metabolic pathways within the body. As metabolic regulators, the farnesoid X receptor (FXR) and the liver X receptor (LXR) play key roles in the pathogenesis of T2D, which remains to be clarified in detail. Here we review the recent progress concerning the physiological and pathophysiological roles of FXRs and LXRs in the regulation of bile acid, lipid and glucose metabolism and the implications in T2D, taking into account that these two nuclear receptors are potential pharmaceutical targets for the treatment of T2D and its complications.

## 1. Introduction

Type 2 diabetes (T2D) is a rapidly growing health concern worldwide, causing serious physical harm and economic burden to the afflicted. According to the World Health Organization (WHO), in 2013, approximately 347 million people globally have diabetes, of which T2D was accounting for around 90%. Moreover, a large-scale study estimates that the world prevalence of diabetes can be up to 7.7% (439 million adults) by 2030 [1], indicating a growing burden to us.

Nuclear receptors (NRs) comprise a superfamily of transcription factors, providing an excellent paradigm for translating extracellular signals into changes in gene expression. Basic NR structure highlights the ligand-binding domain (LBD) and the DNA-binding domain (DBD) [2]. These receptors respond to ligands by up- or downregulating certain signaling pathways with transcriptional control of coactivators and corepressors. Although recent findings are put forth to add to the complexity of the direct and strong classic model of NR function [2], decades of research has suggested that NRs are tractable targets for diabetes therapy, especially FXRs and LXRs, which work in a coordinated fashion to regulate multiple metabolic pathways [3].

This review mainly focuses on the biological roles of the nuclear receptors FXRs and LXRs in the regulation of gene expression as well as metabolism and their changes in T2D. As a result, potential therapeutic strategies linked to their modulation mechanisms may be appreciated to achieve a good glycemic and blood-lipid control in T2D patients.

## 2. Nuclear Sterol-Activated Receptors FXR and LXR

NR superfamily has been classified into seven families: NR1 (thyroid hormone like), NR2 (HNF4-like), NR3 (estrogen like), NR4 (nerve growth factor IB-like), NR5 (fushi tarazu-F1 like), NR6 (germ cell nuclear factor like), and NR0 (knirps or DAX like) [4]. Both farnesoid X receptor *α* (FXR*α*, NR1H4) and liver X receptors (LXRs) *α* and *β* (NR1H3 and 1H2, resp.), which belong to the NR1H subfamily, can respond to steroidal compounds: FXRs respond to bile salts and LXRs to oxysterols (and limited numbers of bile acids).

FXR was first isolated as a mammalian orphan nuclear receptor due to not identifying its ligands, with heterodimeric association to retinoid X receptor [5]. Shortly after its discovery, farnesol derivatives were found to be effective activators, where FXR was originally named after [6]. In 1999, studies revealed that FXR is a transcriptional sensor for bile acids, which are likely the more physiologically important endogenous ligands than farnesol derivatives [7]. In subsequent years, a number of FXR ligands were found, especially several synthetic agonists such as fexaramine, GW4064, and T-0901317 [8]. Besides, FXR is typically expressed at high levels in the liver, intestine, kidney, and adrenal glands [6], while being at low levels in the heart, adipose, and vasculature. In addition, FXR*β* (NR1H5), found in mice, appears to be a nonfunctional pseudogene in humans and other primates and therefore will not be discussed further here [9].

LXRs are so named based on the initial isolation from the liver and liver-rich expression pattern [10]. LXR*α* is detected at high levels in metabolic organs such as liver, adipose tissues, kidney, intestine, and spleen [10]; in contrast, LXR*β* is ubiquitously expressed, the basis for an original name as “ubiquitous receptor” [11]. Although the identification of corresponding ligands nearly twenty years ago [12], synthetic LXR modulators have been the subject of ongoing investigation [13]. Likewise, LXRs function as permissive heterodimers with the retinoid X receptors, with either a LXR ligand or a RXR ligand synergizing to release corepressors and recruit coactivators to regulate the transcription of target genes [10].

## 3. FXR and Metabolism in T2D

### 3.1. FXRs in Bile Acid Metabolism

Bile acids are synthesized from cholesterol by the liver. On one hand, bile acids are essential components of bile and therefore facilitate the digestion and absorption of dietary fats and fat-soluble vitamins after a meal. On the other hand, the biosynthesis of bile acids is a major pathway for removal of cholesterol from the body. Even more important, bile acids have been identified to be natural FXR ligands. As outlined below, on its activation by bile acids, FXR regulates various aspects of lipid and glucose metabolism. Besides, the existence of circulating bile acids is required to some extent for maintaining FXR expression [14]. FXR also regulates bile acids synthesis, conjugation, detoxification, and transport.

95% of the bile acids expelled into the small intestine are reabsorbed from the terminal ileum to complete the enterohepatic circulation; then, the lost part per cycle is compensated for by hepatic synthesis from cholesterol. As bile acids overload is toxic to cells, a feedback loop FXR involved in that inhibits bile acid synthesis is crucial to maintain the bile acid pool size constant. FXR inhibits the conversion from cholesterol to bile acids mainly through suppressing the expression of CYP7A1, the rate-limiting enzyme of the classic pathway. In liver, FXR activation induces expression of the atypical nuclear receptor small heterodimer partner (SHP or NR0B2). SHP, in turn, represses expression of CYP7A1 by inhibiting the activity of liver receptor homologue 1 (LRH-1 or NR5A2, also known as FTF), an orphan nuclear receptor which transactivates CYP7A1 expression [15–17]. Nevertheless, recent studies indicate that LRH-1 is not essential for FXR-mediated feedback regulation of bile acid synthesis [18]. In intestine, FXR activation directly induces expression of fibroblast growth factor-19 (FGF-19) (or FGF-15 in mouse), which subsequently returns to the liver via the portal vein to interact with FGF receptor 4 (FGFR4) in the liver and then downregulates CYP7A1 through a c-Jun N-terminal kinase (JNK) dependent pathway [17, 19, 20]. Simultaneously, FXR also mediates an inhibition of CYP8B1, which controls the ratio of colic acid over CDCA and catalyzes the synthesis of colic acid, by reducing the transactivation activity of the hepatic nuclear factor 4*α* (HNF4*α* or NR2A1) through interaction of SHP with HNF4*α* in the liver [21]. Besides, several studies using liver- and intestine-specific FXR-null models demonstrate that activation of FXR in the intestine but not liver is predominant in mediating feedback repression of bile acid synthesis [17, 22]. In addition to FXR pathways, multiple redundant pathways exist [23–25], and even a few of them are suggested to be more relevant for the negative regulation of CYP7A1 gene transcription [25]. However, abundant evidence indicates that FXR is of vital importance, as deletion of FXR in mice causes marked changes in bile acid homeostasis [26, 27].

Beyond the negative regulation stated above, FXR mediates many other pathways to prevent bile acid-induced liver toxicity. First, FXR positively regulates the enzymes responsible for bile acid conjugation to taurine and glycine, bile acid CoA synthetase (BACS), and bile acid-CoA amino acid N-acetyltransferase (BAAT) [28]. This process occurs to increase their hydrophilicity before bile acids secretion into the bile. Second, FXR induces the expression of genes that encode bile acid-modifying enzymes including CYP3A4 [29], sulfotransferase 2A1 (SULT2A1) [30] and UDP-glucuronosyltransferase 2B4 (UGT2B4) [31], which hydroxylates, sulfidates, and glucuronidates bile acids to protect the liver from toxicity, respectively. Then bile acids are secreted by hepatocytes into the gall bladder. Two ABC transporters involved, which are expressed on the canalicular membrane of hepatocytes: the bile salt export pump (BSEP, also called ABCB11) [32, 33] and the multidrug resistance protein (MDR) (MDR-3 in humans and MDR-2 in mice (ABCC2)) [34], are also FXR targets. Subsequently, bile acids are expelled into the intestine after a meal. Within the intestine, FXR decreases bile acids absorption by indirectly repressing the apical sodium-dependent bile salt transporter (ASBT, also known as IBAT) [35], while the mechanism for FXR-induced IBAT repression is species specific, which involves SHP-mediated interference with transactivation factors on the IBAT promoter LRH-1 in mice [35] and RXR/RAR, or possibly GR, in humans [36]. FXR then promotes bile salts movement from the apical to the basolateral membrane of enterocytes via ileal bile acid-binding protein (IBABP or FABP6) [37] and promotes recycling of bile acids to the liver via organic solute transporters OST*α* and OST*β* [38]. After returning to the liver, bile salts are taken up by hepatocytes. FXR limits hepatic bile salt levels by downregulating the expression of sodium taurocholate cotransporting polypeptide (NTCP) [39, 40] and organic anion transporting polypeptide (OATP) [41].

Over the past decades, a growing body of evidence has shown that bile acid metabolism is altered in T2D patients and animal models [42, 43]. These alterations include changes in bile composition and elevated plasma BA levels. It is worth mentioning that hepatic FXR expression is decreased in diabetic animal models [44], whereas the detailed mechanisms of controlling FXR expression have not been completely known yet. In addition to the mechanism that glucose dose-dependently induces FXR gene expression, hepatocyte nuclear factor-1*α* as well as the cytokines tumor necrosis factor-*α* and interleukin-1*β* has been demonstrated to be involved in regulating FXR expression [45, 46]. Accordingly, alterations in bile acid metabolism in T2D patients may at least partially arise from abnormality of FXR expression. Moreover, as alterations in bile acid composition may cause changes in FXR activation [14], concomitant dysregulation of pathways associated with lipid and glucose metabolism might contribute to the development of T2D and its metabolic complications. Therefore, disorder of bile acid homeostasis is either a cause or a consequence of the metabolic disturbances observed during T2D. In this regard, modulation of bile acid metabolism may improve metabolic symptoms linked to T2D and thus agents such as bile acid sequestrants seem to function in antidiabetic therapy.

### 3.2. FXRs in Triglyceride Metabolism

Development of T2D is associated with increased levels of lipids, particularly triglyceride, which exposes diabetic patients to a higher risk of cardiovascular complications. The dyslipidemic phenotype of the FXR-null mouse provides convincing evidence for FXR as a modulator in the bile acid modulation of lipid metabolism [26]. Most studies to date suggest that FXRs induce decrease in triglyceride levels by regulating the expression of several lipid-modulating proteins. ApoCII, known to promote LPL-mediated triglyceride release from VLDL and chylomicrons and triglyceride hydrolysis into fatty acids, has been identified as a direct downstream target of FXR [47]. On the contrary, the expression of ApoCIII, which behaves differently from ApoCII to inhibit LPL activity, is negatively regulated by FXR [48]. Accordingly, activation of FXR reduces the plasma triglyceride levels. Besides, FXR also induces the expression of PLPT, ApoE, and VLDLR and then control lipid metabolism [49, 50]. Moreover, the above-mentioned effects on lipoprotein catabolism and clearance by FXR lead to reduced plasma cholesterol levels as well.

Regulation of lipogenesis by FXR activation also contributes to lowering triglyceride levels. The expression of sterol-regulatory element-binding protein 1C (SREBP1C), which can stimulate its downstream target genes related to triglyceride and fatty acid synthesis, is downregulated by FXR via the induction of SHP [51]. Nevertheless, another study identified an IR-1 element in the FAS promoter and demonstrated a direct activating role of FXR in the FAS gene [52]. This finding apparently contrasts with the well-established triglyceride-lowering effect of FXR. One hypothesis may account for the discrepancies that the direct activation of FAS by FXR functions to maintain adequate fatty acids for cholesterol esterification, although the mechanistic details remain obscure. Another way of FXR-mediated lowering triglyceride levels depends on enhancement of fatty acid oxidation. This occurs via multiple mechanisms, including upregulation of peroxisome proliferator-activated receptor *α* (PPAR*α*) in human cells [53] and increasing the expression of pyruvate dehydrogenase kinase (PDK4) [54], which promotes utilization of fat versus glucose as a source of fuel. Finally, PPAR*γ* coactivator 1*α* (PGC1*α*) has been shown to regulate triglyceride metabolism by stimulating FXR and concomitantly activating its target genes via PPAR*γ* and HNF4*α* [55]. Overall, in view of the crucial roles of FXR in lipid metabolism, the application of FXR agonists may at least exert a beneficial effect on lowering plasma triglyceride levels, which can also improve symptoms of T2D patients.

### 3.3. FXRs in Glucose Metabolism

Bile acids are endogenous ligands that can activate FXR, while glucose is suggested to be involved in controlling FXR gene expression. Studies demonstrated that glucose induces FXR gene expression in a dose- and time-dependent manner via metabolites of the pentose phosphate pathway, whereas insulin reverses this effect [44].

FXR also acts on glucose metabolism. At present, conflicting results have been reported, but all the findings imply that FXR is essential in the regulation of carbohydrate metabolism. As to the involvement of FXR in the regulation of gluconeogenesis, a previous study showed that FXR agonists stimulated the rate-controlling enzyme phosphoenolpyruvate carboxykinase (PEPCK) mRNA expression and glucose output via FXR-peroxisome proliferator-activated receptor *α*-TRB3 pathway [56]. Another subsequent study reinforced the hypotheses of upregulation of gluconeogenic genes by FXR [57]. By contrast, Ma et al. observed that FXR activation by CA suppressed the expression of multiple genes in the gluconeogenic pathway, including PEPCK, PGC-1*α*, and G-6-Pase [58]. Recent studies have also shown that mouse FGF15 and human FGF19, postprandial hormone induced by FXR, inhibit hepatic gluconeogenesis through a mechanism involving the dephosphorylation and inactivation of the transcription factor cAMP regulatory element binding protein (CREB) [59]. Nevertheless, a recent observation may provide a more reasonable illustration for the discrepancies that FXR activation exerts opposite effects during the transition from the unfed state to the fed state. FXR negatively regulates PEPCK and G6Pase in fed conditions, but it does the opposite in unfed conditions [60]. Besides, an FXR-GR pathway is suggested to regulate the gluconeogenesis [56, 60]. However, in the fasting state, FXR activation is probably weak because bile acids are stored in the gallbladder and are not circulating as in the fed state. Moreover, fasting induces hepatic expression of PGC-1*α* and FXR to generate more energy [55], which adds to the complexity and may stimulate new studies on the mechanism. So gluconeogenesis induced by FXR under this physiological condition needs further investigation. Another recent finding revealed that hepatic expression of aldo-keto reductase 1B7 (Akr1b7), a gene previously linked to detoxification and induced by activated FXR, significantly lowered plasma glucose levels in both wild-type and diabetic db/db mice, for which the inhibition of hepatic gluconeogenesis may be part of the reason [61]. Thus, it will be interesting to investigate whether the closest human ortholog AKR1B10 may be a therapeutic target for diabetes treatment. In addition, data show that bile acids also inhibit PEPCK gene transcription through the farnesoid X receptor-independent mechanism in the context of the fasted-to-fed cycle [25]. Finally, studies on the regulation of glycolysis by FXR have shown relative consistent results. FXR negatively regulates hepatic glycolysis and lipogenesis both in mouse liver and in human hepatocytes, as a result of transrepressing the expression of several glycolytic genes by interference with carbohydrate response element binding protein (ChREBP) transcriptional activity [57, 62].

Indeed, FXR exerts a substantial influence on hepatic carbohydrate metabolism and loss of FXR disrupts normal glucose homeostasis and contributes to the development of insulin resistance [58]. Although the role of FXR in carbohydrate metabolism has been the subject of ongoing investigation, the exact molecular mechanisms are still undoubtedly complex. And it is necessary to reconsider the different effects of bile acids and FXR agonists under fasting or refeeding conditions.

Of note, differences exist under physiological and pathological conditions. Firstly, hepatic FXR expression is decreased in diabetic animal models and normalized upon insulin supplementation [44]. Secondly, FXR activation by the agonist GW4064 or 6-ethyl-chenodeoxycholic acid (6E-CDCA) significantly reduced plasma glucose levels in diabetic mice, which was associated with repression of hepatic gluconeogenic genes and increased hepatic glycogen synthesis and glycogen content [63, 64]. And FXR-null mice exhibited mildly impaired glucose tolerance and insulin sensitivity, while FXR activation reversed insulin resistance. However, recent reports challenged this view, showing that FXR deficiency in obesity improved glucose homeostasis [65]. The study revealed that FXR differentially acted on glucose metabolism in lean and obese conditions and FXR in adipose tissue contributed to the dysregulation of glucose metabolism in obesity. That means the nutritional status of the organism affecting FXR-mediated regulation of glucose homeostasis. Lee and coworkers emphasized the point by analyzing hepatic genome-wide binding sites of FXR in normal and dietary obese mice [66]. The data revealed reduced FXR binding sites in obesity and direct gene repression by FXR. Based on these observations, it will be interesting to broaden our view about the different roles of FXR in carbohydrate metabolism in T2D, compared to normal conditions.

### 3.4. FXRs in Pancreatic *β* Cells

Recent data identified a role of FXR in *β* cell function and a contribution of *β* cell FXR to glucose homeostasis. Chuang et al. reported the expression of nuclear receptors in the endocrine pancreas and found FXR mRNA in mouse islets for the first time [67]. Renga et al. and Popescu et al. further investigated and found the expression of FXR*α* in pancreatic *β*-cells both in rodents and in humans [68, 69]. Several studies consistently suggested that FXR activation increases glucose-stimulated insulin secretion [68–71]. The mechanisms involved seem to include genomic as well as nongenomic effects. First, FXR activation induces the expression of glucose-dependent transcription factor krueppel-like factor 11 (KLF11), which is an essential modulator for insulin gene transcription. Then the nongenomic actions rely on an Akt mediated stimulation of translocation of the glucose transporter 2 (GLUT2) in *β*-cells, which can increase the glucose uptake by cells [68]. Another nongenomic mechanism was found to be relevant to K_ATP_ channel inhibition. The study showed that both sodium taurochenodeoxycholate (TCDC) and FXR agonist GW4064 stimulated the electrical activity of *β*-cells in mice and increased cytosolic Ca^2+^ concentration by inhibiting K_ATP_ current [71]. Thus, FXR activation acutely stimulates insulin secretion. Interestingly, the cellular localization of FXR in *β* cells also depends on the nutritional status of the organism, which is somewhat similar to FXR-mediated regulation of glucose homeostasis as outlined above. Popescu and coworkers reported that FXR localization is predominant in the cytosol of islets in lean mice, but in the nuclei in obese mice [69]. The translocation from the extranuclear space to the nucleus under metabolic stress conditions, for example, insulin resistance or obesity, may account for the phenomenon. However, Renga et al. described an opposite observation that FXR was primarily localized in the nucleus [68]. Since discrepancies exist between their results, more experiments are warranted to examine FXR distribution under distinct conditions. Finally, it is remarkable that FXR activation by FXR agonists protects human islets from lipid-induced metabolic stress [69].

Using insulin-deficient diabetic mice model, in vivo experiments demonstrated that FXR activation ameliorated insulin secretion and delayed development of signs of diabetes, hyperglycemia, and glycosuria [68]. Indeed, FXR agonists might exert action by increasing peripheral insulin sensitivity as well. As T2D is characterized by a combination of peripheral insulin resistance and impaired insulin secretion, the study provides further support for the potential application of the FXR agonists in the prevention of T2D. Certainly, whether the mechanisms above also occur in human *β* cells remains to be detected. However, it is strikingly suggested that FXR activation may inhibit insulin secretion in obese animals, contrary to the effects on *β* cells of lean mice [72]. Therefore, before considering possible clinical use of BAs or FXR agonists, the discrepancies need to be resolved.

## 4. LXR and Metabolism in T2D

### 4.1. LXRs in Bile Acid Metabolism

The cholesterol 7a-hydroxylase (CYP7A1) gene, which encodes the rate-limiting enzyme in the catabolism of cholesterol into bile acids, plays an important role in regulation of bile acid biosynthesis and cholesterol homeostasis. In contrast to the repression of CYP7A1 transcription by FXR, CYP7A1 is transactivated by the oxysterol receptor, LXR*α*. Previous work reported an induced expression of CYP7A1 in rats fed a cholesterol-enriched diet [73]. Subsequently, Lehmann et al. identified a functional binding site for the oxysterol receptor LXR in the promoter region of the rat CYP7A1 gene, and LXR*α* was proved to have a higher affinity than LXR*β* for binding to the CYP7A1 LXRE [74]. Thus excess dietary cholesterol can be eliminated from the body by increased metabolism to bile acids, as a result of LXR*α*-dependent upregulation of CYP7A1. Consistent with these studies, LXR*α* knockout mice fail to induce transcription of CYP7A1 when fed high cholesterol diets, causing changes in bile acid pool size, composition, and cholesterol homeostasis [75]. Overall, LXR*α* acts as a cholesterol sensor and mediates feedforward regulation of CYP7A1 transcription to increase bile acid synthesis and cholesterol excretion when cholesterol accumulates. In addition, LRH-1 is characterized as a competence factor in this process that permits transactivation of CYP7A1promoter by LXR*α* [15].

The mechanism in coordinated control of nuclear receptors FXR and LXR to keep bile acid levels under restraint has been reviewed by Lu et al. [15]. In response to elevated levels of cholesterol, LXRs bind the ligands and upregulate the expression of CYP7A1. After that, de novo bile acids production is increased, which in turn activates FXRs and represses bile acids synthesis via FXR-mediated feedback regulation. The similar conclusion was drawn in another study showing that dose-dependent suppression of CYP7A1 mRNA was seen upon feeding the high-fat diet to animals [76]. This is likely related to the fact that the colic acid present in the diet and the excess bile acids converted from cholesterol exert feedback regulation via FXR. However, in contrast to rodent, this LXR*α*-mediated feedforward regulation does not occur in humans [77, 78], which appears to be a reflection of species specificity. Activation of LXR*α* in humans shares the same effect of FXR on CYP7A1, repressing CYP7A1 expression through a similar induction of SHP [78]. As a result, humans on a diet high in cholesterol might fail to convert excess cholesterol into bile acids and tend to develop hypercholesterolemia.

Another novel and significant function of LXR*α* concerning bile acid detoxification has been noticed. The gene of the major human bile acid-glucuronidating enzyme UGT1A3, which can catalyze bile acid glucuronidation and then contribute to detoxification, has been identified as a positively regulated LXR*α* target with a functional LXR response element in its gene promoter [79]. Besides, activation of LXR*α* confers a female-specific stimulation of expression of bile acid sulfotransferase 2A (Sult2a) [80], which can sulfidates bile acids and promote urinary bile acid elimination. Nevertheless, it is currently unknown whether this beneficial effect confined to female mice is associated with sex hormone. All these observations establish LXR*α* as a crucial regulator of bile acid detoxification to protect the liver from toxicity.

Finally, it is noteworthy that 6a-hydroxy bile acids, products of the acidic bile acid pathway, are identified as another natural ligand of significance for LXR*α* [81]. Since the regulation of CYP7A1 by LXR seems to be species specific and the findings are based on using the response element derived from the rat 7a-hydroxylase promoter, 6a-hydroxy bile acids might behave differently in humans. Nevertheless, the finding of 6a-hydroxy bile acids and analogs may provide further insight into development of selective LXR agonists.

### 4.2. LXRs in Cholesterol Metabolism

Based on the finding of LXRs as cholesterol sensors [12], Peet et al. extended the research on interaction between LXR and cholesterol by using LXR-null mice model and uncovered the importance of LXR for cholesterol metabolism [75]. Collectively, most studies to date demonstrate that LXRs function as sterol sensors to induce expression of genes involved in the regulation of cholesterol and lipid metabolism.

In particular, LXRs control cholesterol homeostasis in the body through promoting reverse cholesterol transport. Present results suggest that LXR activates cholesterol transport via induction of a number of genes encoding cholesterol transporters, apolipoproteins, and lipid metabolizing enzymes. Both cholesterol transporters, ATP-binding cassette transporter A1 (ABCA1) and ABCG1, are identified as LXR targets and upregulated by LXR activation [82–84]. The former plays a key role in the ApoAI-mediated efflux of cholesterol, whereas the latter promotes cholesterol removal in the setting of cholesterol loading via HDL [84, 85]. In addition to the ABC transporters, several apolipoproteins involved in reverse cholesterol transport, including ApoE, ApoC1, ApoC2, and ApoC4, are also transcriptional targets for LXRs [86, 87]. Besides, LXR activation induces the expression of lipid remodelling genes, such as phospholipid transfer protein (PLTP) [88], human cholesterol ester transfer protein (CETP) [89], and lipoprotein lipase (LPL) [90], which are all proposed to facilitate efficient reverse cholesterol transport. Similarly, the intracellular trafficking protein ADP-ribosylation factor-like 7 (ARL7) is also believed to show facilitating effect upon LXR induction [91]. In addition, LXR-dependent induction of two other ABC transporter superfamily members, the ABCG5 and ABCG8, has been reported to be responsible for the decrease of dietary sterol absorption and the increase of sterol excretion [92].

Last but not the least, LXRs augment lipogenesis through transcriptional upregulation of the genes encoding sterol-regulatory element-binding protein 1C (SREBP1C), acetyl CoA carboxylase (ACC), stearoyl CoA desaturase 1 (SCD1), and fatty acid synthase (FAS) [93, 94]. LXRs also regulate the expression of angiopoietin-like protein 3 (Angptl3), leading to hypertriglyceridemia via inhibition of LPL activity and activation of lipolysis in adipocytes [95–97]. Indeed, although LXR activation can bring beneficial effects, the consequence of high triglyceride levels has been the major obstacle in the success of LXR agonists as pharmacologic therapy. Regarding targets for T2D, Chisholm et al. demonstrated an undesirable result of severe lipogenesis on diabetic mouse treated with LXR agonists [98]. Therefore, any agent derived from LXR agonists should be anticipated to lack this side effect before development for T2D treatment.

### 4.3. LXRs in Glucose Metabolism

The role of LXRs in the control of glucose homeostasis in the body has been studied during the past decade. Several studies have suggested that LXR activation has potent serum glucose-lowering effects [99, 100]. Stulnig et al. confirmed that LXR agonist treatment downregulated gluconeogenesis in liver of wild-type mice through a striking decrease in the expression of the key enzymes, namely, PEPCK and G6Pase [101]. Meanwhile, other reports revealed that administration of synthetic LXR agonists significantly reduced blood glucose and improved glucose tolerance in diabetic animal models, owing to coordinate regulation of genes involved in glucose metabolism in liver and adipose tissue [99, 100, 102, 103]. The mechanisms involved in downregulating expression of gluconeogenic enzymes in liver include interaction of LXR with the cofactor receptor-interacting protein 140 (RIP 140) by LXR ligands [104]. Besides, LXR activation also induces the expression of hepatic glucokinase (GK) in liver, which increases glucose flux into the liver and thus enhances glucose utilization [99]. As to the mechanisms in adipose tissue, the antidiabetic effect arises from upregulation of the insulin-sensitive glucose transporter (GLUT4), which promotes glucose uptake and utilization in WAT [99, 105]. Taken together, these effects collectively would be expected to limit hepatic glucose output and promote peripheral glucose uptake, although debate exists as to whether the antidiabetic effects of LXR ligands are primarily due to suppression of gluconeogenesis in liver or not [102, 103]. Perhaps the differences in type of agonists (T-0901317 versus GW-3965) used, dose of administration, treatment condition, and diabetic animal model could account for the discrepancies. However, another study on LXR-deficient mice reported improved metabolic control [106]. This is likely related to the fact that fatty acids interfere with glucose utilization [107]; thus, the reduced level of fatty acids resulted from LXR deficiency contributes to enhanced glucose utilization. Moreover, this is in accordance with the above observation that LXR activation affects blood glucose levels depending on the state of the organism. In any case, it is apparent that LXR agonists lower serum glucose levels only in hyperglycemic state, as the beneficial effects are only seen in diabetic animal models but not in lean mice [102]. Therefore, LXR agonists have been proposed as antidiabetic agents.

Unfortunately, administration of currently available LXR agonists dramatically raises plasma triglyceride levels and induces liver steatosis [98, 102], which are unwanted side effects and have severely hampered the development of the agents. Therefore, a selective modulator that can separate effects of LXR on glucose metabolism from lipogenesis would be useful as antidiabetic agents. Given that LXR*α* is detected at high levels in liver [10], activation of LXR*α* accordingly leads to seriously increasing in lipogenesis. As a result, LXR*β*-selective compounds are thought to act on peripheral tissues and avoid the lipogenic adverse effects [108]. Since induction of GLUT4 expression in adipose tissue is directly mediated by action of LXR on the GLUT4 promoter [105], the development of gene or tissue-selective compounds is essential for diabetes treatment. Interestingly, only LXR*α* affects adipose GLUT4 basal expression and LXR*β* might prevent prolonged LXR*α*-mediated activation [105]. In light of these evidences, adipose-specific activation of LXR*α* might be a novel tool for T2D therapy. Nevertheless, LXR activation seems to inhibit glycolysis in adipose tissues, a clearly undesirable effect under diabetic conditions [101].

However, somewhat unexpectedly, a recent report challenged this view, showing that LXR agonist treatment impaired insulin-mediated glucose uptake in human fat cells derived from overweight individuals [109], which is in contrast to earlier findings in murine models. The authors described the mechanism as suppression of several insulin signalling proteins, namely, Akt2, c-Cbl-associated protein and caveolin-1, and accordingly inhibition of GLUT4 translocation. Despite the so far very limited number of studies performed in human adipocytes and adipose tissue, present results implicated apparent species differences in LXR function. Therefore, more research will be required to determine whether LXR can be considered as a drug target in the treatment of T2D.

### 4.4. LXRs in Pancreatic *β* Cells

Both LXR*α* and LXR*β* isoforms of the receptor are expressed in human and rodent pancreatic islets, whereas LXR*β* is more highly expressed than LXR*α* in insulin-secreting cell lines [67, 110]. Interestingly, like FXR*α*, LXR activation in pancreatic *β*-cells results in enhanced glucose-dependent insulin secretion and expression as well. Numerous lines of evidence have shown that these effects are achieved via regulation of glucose and lipid metabolism after treatment with LXR agonists [110–113]. That is, in addition to the classic K_ATP_-dependent mechanism, other mechanisms like anaplerotic pathways may be involved. Furthermore, Zitzer et al. highlight the functional role of the LXR target gene SREBP-1 in the effects induced by LXR*β* activation [112]. SREBP-1-mediated activation of cataplerosis in *β*-cells is suggested to be one of the mechanisms of LXR*β*-induced insulin secretion. And SREBP-1 is involved to some extent in LXR*β*-upregulated expression of PDX-1, a major transactivator of the insulin gene expression. SREBP-1 also directly binds to three sterol response elements on the insulin gene promoter to activate the insulin gene expression [114]. Consistent with these observations, LXR*β*−/− mice are glucose intolerant due to impaired glucose-induced insulin secretion by LXR*β*−/− islets, instead of reduced insulin sensitivity [115].

All these data suggest a positive role of LXR in *β*-cells in coupling of glucose metabolism to insulin secretion; in spite of this, LXRs have been proved to be involved in growth arrest and apoptosis in pancreatic *β*-cells [116–119]. Wente et al. reported that activation of LXR/RXR heterodimers by a combination of the LXR agonist T0901317 with the RXR agonist 9-*cis-*retinoic acid (9cRA) inhibited proliferation and induced apoptosis in MIN6 cells and isolated rat islets, though T0901317 showed no effect on proliferation of MIN6 cells by itself [116]. The authors demonstrated that activation of LXR/RXR stimulated expression of mothers against decapentaplegic homolog 3 (Smad3), a protein known to prevent cells from G1 to G2 phase progression and promote TGF-*β*-induced apoptosis on its overexpression [120, 121]. Another study observed inhibition of pancreatic beta cell proliferation by LXR agonists alone. As a result of upregulation of p27 protein, a regulator of beta cell cycle progression, T1317 dose-dependently induced growth inhibition through cell cycle arrest in hamster HIT-T15 cells, mouse MIN6 cells, and isolated mouse islets [117]. Besides, LXR activation by T0901317 significantly promoted palmitic acid-induced apoptosis in pancreatic *β*-cells by aggravating effects of palmitic acid on caspase activity [116, 118]. Moreover, activation of LXR in INS-1 cells and rat islets induces lipogenic gene expression, which leads to intraislet lipid accumulation and eventually *β*-cell failure if prolonged LXR activation exists [119, 122].

These results are likely related to the fact that LXRs in pancreatic *β*-cells can exert different effects on the cells depending on the level and duration of LXR activation [110, 116, 119], thus indicating that appropriate regulation of LXR activity is beneficial for *β* cell function. Considering this fact and the species-specific difference in the response to agonists [123], more studies are needed to clarify the dose-dependent and time-dependent effects of LXR agonists in vivo before these agents are developed for clinical application. However, although appropriate pharmacologic activation of LXRs brings favorable changes in metabolism as well as in islet function, the adverse effect of hepatic steatosis that resulted from upregulation of lipid biosynthesis has been the limitation to the development of LXR agonists for human use.

Given that LXR functions in a regulated and controlled manner under physiological conditions, the alteration of LXR activation in *β*-cells during environmental changes might be involved in diabetes pathogenesis. The number of pancreatic *β*-cells in the body is controlled by the balance between processes of proliferation and apoptosis. Elevated *β*-cell apoptosis has been implicated as an important mechanism for the decrease in the *β*-cell number in type 2 diabetes [119, 124, 125]. However, except for amyloid deposits derived from IAPP (islet amyloid polypeptide) [124, 126, 127], chronic LXR dysregulation occurs in the disease state and contributes to the *β*-cell dysfunction in T2D. Previous studies reported that islets from diabetic animals displayed increased levels of SREBP-1 [128], one of LXR target genes. Choe et al. have demonstrated that both LXR*α* and LXR*β* mRNA levels are apparently elevated in the islets of diabetic animal models [119]. More importantly, they revealed that the high blood glucose condition of diabetic subjects would aggravate the chronic LXR activation-induced lipotoxicity and *β*-cell apoptosis. In addition, Helleboid-Chapman et al. reported that glucose had no effect on LXR*α* protein level but regulated its subcellular localization in INS-1 cells [113]. They showed that addition of glucose caused translocation of LXR*α* from the cytoplasm to the nucleus, thus resulting in a higher transcriptional activity of LXR*α* upon treatment with LXR agonists, whereas there was no data about LXR*β*. Moreover, plasma levels of oxysterols, endogenous ligands for LXRs, are greatly increased in diabetic patients [129]. All these data suggest that aberrant expression and activation of LXRs in *β*-cells may contribute to type 2 diabetes development to a certain extent. However, no quantitative data have been reported so far and therefore more studies are needed to determine the complicated mechanisms.

## 5. Latest Advances

At present, the nuclear receptors are considered as important drug targets due to their regulation roles in both physiological and pathological processes. Given that FXRs and LXRs modulate many metabolic pathways and are intimately linked to development of type 2 diabetes, they have emerged to be promising therapeutic targets for T2D.

Bile acid sequestrants (BAS), which have been used for control of hyperlipidaemia for decades, have been proven to ameliorate hyperglycemia in T2D patients. In particular, colesevelam has already received an indication from the US FDA for lowering glucose in patients with type 2 diabetes. The glucose-lowering effect of BAS on humans was first demonstrated two decades ago [130]. Subsequently, several clinical trials were performed to further testify the effects [131, 132]. The mechanisms involved are complex and not well established. BAS bind bile acids in the intestine, thus reducing the endogenous ligands for FXR and therefore acting to reduce FXR activation. In turn, downregulation of gluconeogenetic gene is induced. Certainly, one must keep in mind that bile acids are also ligands for TGR5 and therefore TGR5-mediated suppression of glycogenolysis is essential [133]. However, by attenuating FXR activation, BAS impair triglyceride metabolism, which presents as side effects.

Surprisingly, apical sodium-dependent bile acid transporter inhibitor has recently emerged as a novel treatment for diabetes [134, 135]. The inhibitors function by blocking the reuptake of bile acids in the ileum and upregulating hepatic bile-acid synthesis. As bile acids are also ligands for other nuclear receptors, just like BAS, the mechanisms concerning bile acids signaling are obscure. Factually, FXR activity is decreased upon Asbt inhibitor administration [134].

FXR agonists are likely to be effective triglyceride-lowering drugs and they may also have potential benefits in reducing elevated glucose. Recently, studies have suggested that FXR agonist obeticholic acid (INT-747) might be applied in T2D treatment. In a phase 2 clinical trial, administration of OCA showed increased insulin sensitivity [136]. Ghebremariam et al. demonstrated that INT-747 upregulated the expression of liver DDAH1, which has a FXR binding site on its promoter, and then enhanced insulin sensitivity in Dahl rats [137]. However, clinical applications of FXR agonists remain much less developed; thus, more clinical studies on FXR agonists will be necessary to identify their roles.

What is more, the antiparasitic drug ivermectin has been identified as a novel ligand for FXR recently [138]. The study reported that ivermectin treatment can reduce serum glucose and cholesterol levels by directly targeting FXR. Since it has already been a clinical drug approved by the FDA, ivermectin may be put into rapid clinical applications with desirable therapeutic potentials.

In addition, cyanidin, a natural flavonoid, is found to be an agonistic ligand for LXR. It can bring an effect of reducing cellular lipid accumulation in macrophages and hepatocytes [139, 140]. As dietary regulators of LXR activity, flavonoids may get a good application. Besides, genetic variations in LXR have been investigated and the studies revealed that rs17373080 in LXR*β* may be part of the pathogenesis of T2D, as a result of altered LXR*β* expression [141, 142]. However, the first published experiment with a synthetic LXR agonist (LXR-623) in humans was prematurely stopped due to central-nervous system-related adverse effect [143]. Last but not least, as LXR and FXR are almost ubiquitously expressed, they have been involved in testis [144] and adrenal [145] physiologies, thus being associated to reproduction [146, 147]. Therefore, numerous adverse effects of LXR activation need to be solved before genuine clinical trials in humans.

## 6. Conclusions and Perspectives

**Figure 1:**
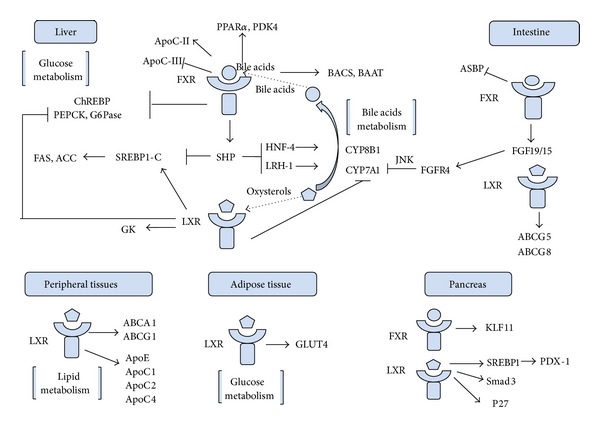
FXR and LXR target genes in bile acids, lipid, and glucose metabolism.

Coordinated actions of nuclear receptors FXR and LXR exert a complex role in transcriptional regulation of genes involved in bile acid, lipid, carbohydrate metabolism, and pancreatic *β*-cell function (Figure 1). Present studies have indicated that they are both associated with the development of T2D. Therefore, FXR and LXR represent promising therapeutic targets for diabetes drug discovery. However, future advances in this field are needed to clarify the tissue-specific differences of the FXR and LXR pathways in order to develop agents with selectivity and avoid potential side effects. Moreover, the changes of FXR and LXR expression in T2D patients still lack research. And whether the alteration of their expression is primarily the cause or result of the metabolic disorders in T2D might repay investigation. Certainly, the understanding of the pathogenesis of T2D is continuing to evolve.
